# Aspirin before the 11 ${\;}^{\text{th}}$ week of pregnancy to prevent pre-eclampsia

**DOI:** 10.18502/ijrm.v22i8.17245

**Published:** 2024-10-14

**Authors:** Marie-Laurence Côté, Brielle Demuth, Louise Ghesquière, Emmanuel Bujold

**Affiliations:** ^1^Research Center of CHU de Québec-Université Laval, Québec, Qc, Canada.; ^2^CHU de Lille, Université de Lille, Lille, France.; ^3^Department of Obstetrics and Gynecology, Faculty of Medicine, Université Laval, Québec, Qc, Canada.

##  Dear Editor,

It is with great interest that we read the research protocol published by the team of Hantoushzadeh et al. (1). The proposed randomized trial aims to answer a very important question in the field of obstetrics: What is the optimal gestational age for starting aspirin to prevent pre-eclampsia? Indeed, the publication of several meta-analyses and a multicenter randomized trial demonstrated that aspirin started at the end of the 1 ${\;}^{\text{st}}$ trimester (between 11 and 16 wk gestation) reduced the risk of preterm pre-eclampsia and other placenta-mediated complications in high-risk women. The use of aspirin has become increasingly common, with the belief that the earlier the aspirin is started, the more effective it is (2–4). However, the authors of this study have clearly stated that currently there are no studies that have adequately investigated starting aspirin in the early weeks of pregnancy (before the 9 ${\;}^{\text{th}}$ or 10 ${\;}^{\text{th}}$ wk), and a meta-analysis of small randomized trials concluded that starting aspirin before the 11 ${\;}^{\text{th}}$ wk does not better prevent pre-eclampsia (5). The largest study on the subject by Hoffman et al., involved commencing aspirin on average at 10 wk and 1 day (6).

The mechanism by which aspirin reduces the risk of preterm pre-eclampsia and other complications is not well understood. However, we strongly suspect that it improves invasion and transformation of the spiral arteries by cytotrophoblastic cells, which is typically reduced or absent in almost all cases of preterm pre-eclampsia (7). In the Aspirin versus Placebo in Pregnancies at High Risk for Preterm Preeclampsia trial, aspirin at 150 mg daily was shown to be effective in improving uterine artery pulsatility index in the second trimester (3). Moreover, the uterine pulsatility index and placental growth factor (PlGF) are the 2 most important markers for estimating the risk of pre-eclampsia in the 1 ${\;}^{\text{st}}$ trimester of pregnancy. In this context, we believe it is very important to measure these 2 markers at the 11–13 wk visit, to verify whether or not these 2 markers are improved by early aspirin intake. Pregnancy-associated plasma protein-A is a less reliable marker than PlGF for assessing deep placental dysfunction (8).

Finally, previous studies have demonstrated that platelet aggregation tests, notably the Platelet Function Testing-100 test, determine which patients respond best to aspirin, thus identifying those who require a dose greater than 80 mg (9). In the absence of such a test, if a study fails to demonstrate a significant impact of aspirin commenced before week 11, a major trial may still raise unanswered questions, such as could aspirin at a dose greater than 80 mg have a beneficial effect? There is a growing body of scientific evidence suggesting that the prevention of pre-eclampsia with aspirin requires a dose of at least 100 mg per day, or even 150–160 mg (10–12). We still do not know how to identify women for whom a dose of 80 mg a day will be sufficient, but the current trend is to start a dose of 150 or 160 mg at the 11–13 wk visit when the patient is identified as being at risk. Moreover, the protocol proposed by the authors recommends increasing the dose to 160 mg per day if the patient is at high risk at the 11–13 wk visit, a definite strength of the study.

In summary, we are extremely pleased that the proposed trial has gone ahead. However, we believe that to fully benefit from the project, it would be important for platelet aggregation to be assessed by Platelet Function Testing 100 at the 11–13 wk visit, along with PlGF measurement. It would be possible for the beneficial effect to be observed only in patients whose platelet aggregation is modified, bearing in mind that 30–60% of pregnant women may be “resistant" to a dose of 80 mg.

Once again, we congratulate Hantoushzadeh et al., on their project, which has the potential to significantly improve the health of women and children worldwide.

##  Conflict of Interest

None of the authors disclosed any conflict of interest.

## References

[bib1] Hantoushzadeh S, Behzadian A, Hasheminejad MM, Hasheminejad F, Helal Birjandi A, Akbari M, et al (2024). Aspirin administration from early pregnancy versus initiation after 11 weeks of gestation for prevention of pre-eclampsia in high-risk pregnant women: Study protocol for randomized controlled trial. Int J Reprod BioMed.

[bib2] Bujold E, Roberge S, Lacasse Y, Bureau M, Audibert F, Marcoux S, et al (2010). Prevention of preeclampsia and intrauterine growth restriction with aspirin started in early pregnancy: A meta-analysis. Obstet Gynecol.

[bib3] Rolnik DL, Wright D, Poon LC, O'Gorman N, Syngelaki A, de Paco Matallana C, et al (2017). Aspirin versus placebo in pregnancies at high risk for preterm preeclampsia. N Engl J Med.

[bib4] Ghesquiere L, Vachon-Marceau C, Kingdom JC, Ferreira E, Côté S, Guerby P, et al

[bib5] Chaemsaithong P, Cuenca-Gomez D, Plana MN, Gil MM, Poon LC

[bib6] Hoffman MK, Goudar SS, Kodkany BS, Metgud M, Manjunath Somannavar M, Okitawutshu J, et al (2020). Low-dose aspirin for the prevention of preterm delivery in nulliparous women with a singleton pregnancy (ASPIRIN): A randomised, double-blind, placebo-controlled trial. Lancet.

[bib7] Ogge G, Chaiworapongsa T, Romero R, Hussein Y, Kusanovic JP, Yeo L, et al (2011). Placental lesions associated with maternal underperfusion are more frequent in early-onset than in late-onset preeclampsia. J Perinat Med.

[bib8] Wright D, Tan MY, O’Gorman N, Syngelaki A, Nicolaides KH (2022). Serum PlGF compared with PAPP-A in first trimester screening for preterm pre-eclampsia: Adjusting for the effect of aspirin treatment. BJOG.

[bib9] Rey E, Rivard GE

[bib10] Ghesquiere L, Guerby P, Marchant I, Kumar N, Zare M, Foisy MA, et al (2023). Comparing aspirin 75 to 81 mg vs 150 to 162 mg for prevention of preterm preeclampsia: Systematic review and meta-analysis. Am J Obstet Gynecol MFM.

[bib11] Demuth B, Pellan A, Boutin A, Bujold E, Ghesquière L (2024). Aspirin at 75 to 81 mg daily for the prevention of preterm pre-eclampsia: Systematic review and meta-analysis. J Clin Med.

[bib12] Roberge S, Nicolaides K, Demers S, Hyett J, Chaillet N, Bujold E (2017). The role of aspirin dose on the prevention of preeclampsia and fetal growth restriction: Systematic review and meta-analysis. Am J Obstet Gynecol.

